# Associations between conformity to masculine norms and depression: age effects from a population study of Australian men

**DOI:** 10.1186/s40359-021-00533-6

**Published:** 2021-02-19

**Authors:** Danielle Herreen, Simon Rice, Dianne Currier, Marisa Schlichthorst, Ian Zajac

**Affiliations:** 1 School of Psychology, University of Adelaide, Adelaide, SA 5000 Australia; 2 Health & Biosecurity, Commonwealth Scientific & Industrial Research Organisation (CSIRO), Adelaide, SA 5000 Australia; 3 Orygen, Parkville, VIC 3052 Australia; 4 Centre for Youth Mental Health, The University of Melbourne, Melbourne, VIC 3052 Australia; 5 Centre for Mental Health, Melbourne School of Population and Global Health, The University of Melbourne, Melbourne, VIC 3010 Australia

**Keywords:** Depression, Masculinity, Mental health, Ageing, Lifespan

## Abstract

**Background:**

Strict adherence to masculine norms has been associated with deleterious consequences for the physical and mental health of men. However, population-based research is lacking, and it remains unclear whether ageing influences adherence to masculine norms and the extent to which mental health problems like depression are implicated.

**Methods:**

This study reports on data from 14,516 males aged 15–55 years who participated in Wave 1 of the Australian Longitudinal Study of Male Health (Ten to Men). Group differences in self-reported conformity to masculine norms (CMNI-22), current depressive symptoms (PHQ-9), and self-reported 12-month depression history were examined for males aged 15–17 years, 18–25 years, 26–35 years, 36–50 years, and 51–55 years. Generalised linear models were used to examine the relationships between these variables across age groups.

**Results:**

Conformity to masculine norms decreased significantly with age. However, models predicting depression generally showed that higher conformity to masculine norms was associated with an increased risk of current depressive symptoms, especially in the oldest age group. Conversely, higher conformity was associated with a decreased likelihood of a self-reported 12-month depression history, although nuances were present between age groups, such that this trend was not evident in the oldest age group.

**Conclusions:**

Findings provide important insights into the complex relationship between conformity to masculine norms and depressive symptoms across the lifespan and further highlight the importance of mental health campaigns that address the complexities of gendered help-seeking behaviour for men.

**Supplementary Information:**

The online version contains supplementary material available at 10.1186/s40359-021-00533-6.

## Background

Depression is a common mental health problem that engenders significant challenges for the individual, their families, and healthcare systems worldwide [1]. Although women are diagnosed with depression twice as often as men [2], men experience higher rates of substance abuse and dependence [3], and are three times more likely to die by suicide [4]. There is considerable evidence that strict adherence to masculine norms—the socioculturally prescribed expectations and standards of what it means to be a man—are associated with poorer mental health outcomes and reduced help-seeking amongst men [5, 6]. In a Western context, males are often socialised in ways that reinforce norms of self-reliance, stoicism, and avoidance of negative emotions [7]. While these characteristics can be adaptive and result in economic and social benefits to men [8], they can also contribute to cognitions and behaviours that increase the risk of mental health problems including depression [9]. Despite increased research interest in the relationship between masculine norms and mental health in recent years, it remains unclear how, if at all, age influences adherence to masculine norms and whether experiences of depression are implicated.

Age is often acknowledged as an important factor throughout the masculinity literature [10], yet there remains a paucity of research extending beyond early adulthood, particularly in the context of mental health. From a developmental perspective, gendered expectations become especially salient to youth during adolescence and throughout emerging adulthood [11]. During this stage of life, social acceptance is central to behaviour, illustrated by heightened pressure to adhere to stereotypical gender roles [12, 13]. In contrast, theory and research indicate less rigid gender roles throughout the middle and older years [14]. Ageing is associated with a loss of functions that, to varying degrees, contradict the standards of masculinity (i.e., beliefs such as men should be physically strong) [15]. Some research suggests that older men may reconstruct their masculine beliefs to less constraining ones in response to life changes [16]. Other studies suggest, however, that some men struggle to reframe their masculinity attitudes with ageing [15, 17] and continue to view their health and its deterioration through a masculine lens [18]. As such, men with a strict, inflexible adherence to traditional masculine norms may face increasingly negative consequences through ageing as they attempt to conserve their masculinity, for example, by avoiding professional help [19].

One of the most frequently used measures for assessing adherence to masculine norms is the Conformity to Masculine Norms Inventory (CMNI) [20]. While limited research has considered how conformity to masculine norms changes across the lifespan, some studies have shown that younger adults report higher CMNI scores compared to older adults [15, 21]. When considering adherence to specific masculine norms (i.e., CMNI subscales), younger adults have been found to score higher on factors assessing violence, risk-taking, and winning, whilst middle-aged and older adults scored higher on measures of self-reliance and emotional control [22]. Even fewer studies have considered associations between masculine norms and depression across the lifespan. In an attempt to overcome this gap, Rice and colleagues (2011) analysed two samples of Australian males aged 18–64 and found that whilst CMNI scores decreased with age, the strength of its relationship with depression actually increased with age [23]. Notwithstanding this contribution, the study by Rice and colleagues used an online, non-representative sample and it failed to consider the relationship between specific masculine norms and depression, nor the impact of age on these relationships.

Given the compelling evidence that masculinity-related constructs are associated with men’s psychological health [5], and the theorised benefits of addressing gender role conflict throughout the lifespan [24], further research is needed to improve our understanding of how age differences may impact on the relationship between masculine norms, specific domains of masculinity, and measures of depression. Accordingly, this study utilised data from the baseline wave of Ten to Men: The Australian Longitudinal Study on Male Health [25]. Based on the research above, it was hypothesised that younger males would report higher conformity to masculine norms compared to older age groups. In addition, it was hypothesised that specific masculine norms would be differentially related to depressive symptoms and that the strength of these relationships would vary across age groups. Specifically, given recent meta-analytic findings suggesting that self-reliance may be the strongest predictor of poorer mental health outcomes (e.g., [5]), it was hypothesised that self-reliance would be associated with increased depressive symptoms. Finally, we expected that higher conformity to masculine norms would predict an increased likelihood of reporting clinically significant depressive symptoms, as well as a self-reported 12-month depression history in all age groups.

## Methods

### Data source

The study population consisted of 14,516 males aged 15–55 years who participated in the baseline wave (Wave 1) of the Australian Longitudinal Study on Male Health (Ten to Men). Ten to Men is the largest, all-male, national cohort study devoted to male health to be conducted internationally to date [25]. Further information regarding the study design and methodology used in Ten to Men is available elsewhere (see [26]). A brief summary is provided here.

Recruitment and data collection for Wave 1 took place between October 2013 and July 2014 across Australia using a multi-stage stratified cluster random sampling strategy, where the primary unit of sampling was the household. Eligible participants were males aged 10–55 years who resided within private dwellings in the selected sampling regions, were proficient in English, and were Australian citizens or permanent residents. Participants provided information around five broad domains relevant to male health (i.e., physical health, mental health and wellbeing, social determinants of health, health-related behaviours, and health service utilisation and health knowledge) via a self-report paper questionnaire. The conduct of Wave 1 was approved by the University of Melbourne Health Sciences Human Ethics Sub-Committee (HREC 1237897 and 1237376) and conformed to the principles embodied in the Declaration of Helsinki. Participants aged 18–55 years provided written consent, and participants aged 17 or younger provided written assent and a parent/guardian provided written consent.

### Outcomes

#### Current depressive symptoms

Current depressive symptoms were assessed by the Patient Health Questionnaire (PHQ-9) [27]. The PHQ-9 is a validated depression screening tool and is frequently used in both research and clinical practice. The PHQ-9 corresponds to DSM-5 diagnostic criteria for major depressive disorder [28] and assesses nine symptoms present over the preceding two week period (e.g., “*Feeling down, depressed, or hopeless*”). Participants rate their responses on a 4-point Likert scale ranging from 0 (*not at all*) to 3 (*almost every day*). For males aged 15–17 years, an adolescent-tailored version of the same instrument was used which includes an item assessing irritability per DSM-5 criteria for respondents < 18 years (PHQ-A) [29]. Scores on the PHQ-9 and PHQ-A were combined to create a total PHQ-9 score for the sample. Cronbach’s alpha in the present study was 0.86 for the adolescent version and 0.88 for the adult version, demonstrating good internal consistency. The PHQ-9 has been shown to have high sensitivity and specificity for detecting major depressive disorders, with a score of 10 and above indicative of clinically significant depressive symptoms [30]. Higher scores reflect greater severity of depressive symptoms.

#### Self-reported 12-month depression history

Self-reported 12-month depression history was ascertained using a single question. Participants were asked: “*Have you been treated for or had any symptoms of this condition in the past 12 months?*”. This question format was derived from the Australian Health Survey [31] and provides a more objective measure of depression.

### Predictor variables

#### Conformity to masculine norms

Conformity to masculine norms were assessed by the short form of the Conformity to Masculine Norms Inventory (CMNI-22) [20, 32]. The CMNI-22 is an abbreviated version of the original 94-item instrument which was designed to measure the extent to which individuals conform to masculine norms dominant in Western culture (e.g., “*It bothers me when I have to ask for help*”). Participants are asked to think about their own actions, feelings, and beliefs and indicate their level of agreement with each of 22 statements which are scored on a 4-point Likert scale ranging from 0 (*strongly disagree*) to 3 (*strongly agree*).

The CMNI-22 consists of the two highest loading items for each of the original 11 factors to yield factor scores (i.e., the sum of each pair of items) and a total masculinity score. The 11 factors form subscales of the CMNI and include: (1) work; (2) risk-taking; (3) dominance; (4) heterosexual presentation; (5) emotional control; (6) winning; (7) power over women; (8) pursuit of status; (9) violence; (10) playboy; and (11) self-reliance. In the current study, Cronbach’s alpha was 0.66 for the total scale. This is consistent with previous studies (e.g., [33]) and is within the generally accepted range [34]. Higher CMNI-22 scores reflect greater conformity to masculine norms.

#### Age

For statistical modelling, participants were assigned to age group categories consistent with those utilised by Rice and colleagues [23]. The categories were chosen to reflect developmentally distinct groups [35] and were as follows: 15–17 years, 18–25 years, 26–35 years, 36–50 years, and 51–55 years.

### Covariates

We accounted for the influence of two socio-demographic covariates that could contribute to our variables of interest. Participant’s socio-economic status was determined on the basis of their area of residence, using percentages from the Index of Relative Socioeconomic Disadvantage (IRSD) [36]. Lower IRSD scores indicate relatively greater disadvantage. Region of residence was defined according to the Remoteness Area classification of the Australian Statistical Geography Standard (ASGS), and comprised major cities, inner regional areas, and outer regional areas [37]. These covariates were included as main effects in all statistical models, but their influence was considered out-of-scope and therefore results are not reported for brevity.

### Statistical analyses

The present study focused on males aged 15–55 years (*N* = 14,916) who participated in Wave 1 of the Ten to Men study. Participants who completed less than 80% of the CMNI-22 and PHQ-9 (*n* = 398) were excluded from the dataset. Data for a further two individuals regarding their ASGS region was missing and they were also excluded. The refined sample for analytic purposes was *N* = 14,516 cases. Existing recommendations relating to the use of TTM data indicate weighting is necessary for estimating population parameters. However, the requirement to weight data is largely redundant in the instance of estimating associations [38]. Therefore, given the present aim was to examine relationships between age, masculine norms, and measures of depression, data were not weighted for the analyses described herein. To facilitate scoring of the CMNI-22 and PHQ-9, missing item-level data for these scales were replaced with the intra-individual mean for that scale. Participants were then assigned to age group categories as described above. Participants were also assigned to CMNI categories using the approach described by Mahalik et al. by converting raw scores to transformed scores [38]. Four categories were constructed to reflect extreme non-conformity to masculine norms, moderate non-conformity, moderate conformity, and extreme conformity. In addition to examining PHQ-9 total scores, we created a binary variable with scores ≥ 10 indicating clinically significant depressive symptoms, consistent with published cut-off scores for the PHQ-9 [30].

Generalised linear models (GLMs) using maximum likelihood estimation were conducted to examine the relationships between variables. Model assumptions were found to be upheld by inspection of scatter plots and histograms of residuals and predicted values. For the model examining the relationship between CMNI factors and current depressive symptoms measured using the PHQ-9, we used a Guassian distribution. A backwards elimination method (*p* > 0.05) was employed to iteratively remove non-significant CMNI factor by age interactions to arrive at the final model. For this model with PHQ-9 total score as the dependent variable, results are reported as standardised betas. For models with categorical outcomes (i.e., CMNI category and 12-month depression history), a binary distribution and logit link function was used, and odds ratios are reported. For age group interactions, the reference category used was 51–55 years, while for CMNI interactions, the reference category was extreme non-conformity. All models were adjusted for the covariates described above and data were analysed using SPSS (Version 26.0).

## Results

Descriptive statistics for the overall sample are presented in Table 1. The majority of the sample resided in metropolitan areas, were born in Australia, and were of heterosexual orientation. Around 1 in 10 participants reported clinically significant depressive symptoms (PHQ-9 ≥ 10) or a 12-month history of depression. Comparisons of the TTM cohort with the Australian male population are reported elsewhere (see [39]) and highlight that despite some minor differences, the TTM sample mirrors the general population reasonably well.

**Table 1:** Sociodemographic characteristics for the overall sample

	N	%
Age categories (M, *SD*)		
15–17 years (15.98, *0.81*)	986	6.8
18–25 years (21.42, *2.31*)	2196	15.1
26–35 years (30.81, *2.86*)	3141	21.6
36–50 years (43.15, *4.27*)	6205	42.8
51–55 years (52.86, *1.33*)	1988	13.7
Region		
Major cities	8559	59.0
Inner regional areas	3222	22.2
Outer regional areas	2735	18.8
Country of birth		
Australia	11,179	77.0
Overseas	3294	22.7
Did not report	43	0.3
Sexuality		
Heterosexual	13,190	90.9
Bisexual/homosexual/other	652	4.5
Not sure	311	2.1
Did not report	363	2.5
Education^a^		
Pre year 12	1826	13.5
Year 12	2000	14.8
Certificate/diploma	6136	45.3
Undergraduate degree	2092	15.5
Postgraduate degree	1241	9.2
Did not report	235	1.7
Employment status^a^		
Employed	11,472	84.8
Unemployed	1115	8.2
Not in labour force	815	6.0
Did not report	128	0.9
Marital status^a^		
Never married	3510	25.9
Widowed/divorced/separated	883	6.5
Married/de-facto	9012	66.6
Did not report	125	0.9
Clinically significant depressive symptoms (PHQ-9 ≥ 10)	1905	13.1
12-month depression history	1822	12.6

^a^Participants aged 15–17 were not asked this question

**Fig. 1:**
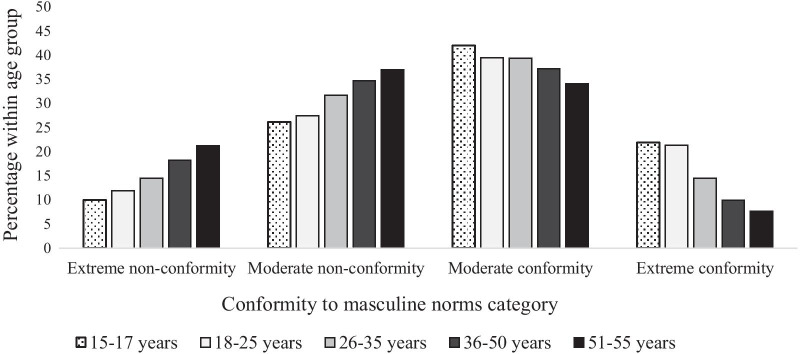
Conformity to masculine norms category by age group

The likelihood of being in the extreme conformity to masculine norms category decreased with age (see Fig. 1). Chi-square analysis supported this trend, χ^2^(12) = 433.87, *p* < 0.001, with significantly higher than expected numbers of participants in the extreme non-conformity and moderate non-conformity categories in older age groups.

Next, we explored the effect of each of the 11 conformity to masculine norms inventory (CMNI) factors (i.e., subscales) on depressive symptoms (PHQ-9 total score) including their interactions with age. Descriptive statistics for CMNI factors for each age group are available in the Additional file 1: Table S1 accompanying this paper. Results of the final multivariable model are shown in Table 2. Age was a significant predictor of current depressive symptoms with those aged 51–55 having lower mean PHQ-9 scores than the 15–17, 18–25, and 26–35 year-old groups. In terms of CMNI factors, work, dominance, risk-taking, heterosexual presentation, violence, and status were all significant predictors of current depressive symptoms and this did not differ across age groups. Of these, only violence was significantly positively related to depressive symptoms, whilst the other factors were inversely related (i.e., higher levels were associated with less depressive symptoms). Significant interaction effects with age existed for playboy, power over women, self-reliance, and winning. The influence of playboy on depressive symptoms was significantly stronger in all age groups relative to those aged 51–55 years. In contrast, winning had a significantly stronger influence on depressive symptoms in the 51–55 group compared to all others. Self-reliance was also more strongly linked to depressive symptoms in those aged 51–55 years compared to all groups other than those aged 18–25 years. Finally, power over women was more strongly related to depressive symptoms in the 51–55 group relative to those aged 15–17 and 18–25 years but was comparable to those aged 26–35 and 36–50.

**Table 2:** Current depressive symptoms (standardised PHQ-9 scores) predicted by age and conformity to masculine norms (standardised CMNI-22 factors)

	β^b^	*p* value	95% CI
(Intercept)	0.11	**0.00**	[0.05, 0.17]
Age^a^			
15–17	0.08	**0.03**	[0.01, 0.16]
18–25	0.14	**0.00**	[0.08, 0.20]
26–35	0.09	**0.00**	[0.03, 0.14]
36–50	0.05	0.06	[0.00, 0.10]
CMNI factors			
Work	− 0.05	**0.00**	[− 0.06, − 0.03]
Playboy	0.01	0.60	[− 0.03, 0.06]
Winning	0.08	**0.00**	[0.03, 0.13]
Self-reliance	0.33	**0.00**	[0.29, 0.38]
Dominance	− 0.03	**0.00**	[− 0.04, − 0.01]
Risk-taking	− 0.03	**0.00**	[− 0.05, − 0.02]
Emotional control	− 0.01	0.36	[− 0.02, 0.01]
Heterosexual presentation	− 0.02	**0.03**	[− 0.04, 0.00]
Power over women	0.00	0.87	[− 0.05, 0.04]
Violence	0.05	**0.00**	[0.03, 0.07]
Status	− 0.02	**0.01**	[− 0.04, − 0.01]
Age × playboy^a^			
15–17	0.11	**0.00**	[0.04, 0.19]
18–25	0.07	**0.02**	[0.01, 0.13]
26–35	0.12	**0.00**	[0.06, 0.17]
36–50	0.06	**0.04**	[0.00, 0.11]
Age × winning^a^			
15–17	− 0.09	**0.01**	[− 0.16, − 0.02]
18–25	− 0.12	**0.00**	[− 0.18, − 0.06]
26–35	− 0.11	**0.00**	[− 0.17, − 0.05]
36–50	− 0.08	**0.00**	[− 0.14, − 0.03]
Age × self-reliance^a^			
15–17	− 0.09	**0.02**	[− 0.16, − 0.02]
18–25	− 0.03	0.26	[− 0.09, 0.02]
26–35	− 0.10	**0.00**	[− 0.15, − 0.04]
36–50	− 0.06	**0.02**	[− 0.11, − 0.01]
Age × power over women^a^			
15–17	− 0.09	**0.02**	[− 0.16, − 0.01]
18–25	− 0.07	**0.03**	[− 0.13, − 0.01]
26–35	− 0.01	0.70	[− 0.07, 0.05]
36–50	− 0.02	0.55	[− 0.07, − 0.04]

Bold values denote statistical significance at the* p* < 0.05 level

All models adjusted for Index of Relative Social Disadvantage (IRSD) and Remoteness Area

^a^Reference group for age is the 51–55 age group

^b^Interaction terms represent the difference in β’s between the comparison group and reference group

The interaction between age, CMNI category, and different indices of depression (i.e., PHQ ≥ 10, self-reported 12-month depression history) are presented in Table 3. Extreme conformity to masculine norms was associated with a higher likelihood of reporting clinically significant depressive symptoms but only in the 36–50, and 51–55 age groups. The relationship between CMNI category and self-reported 12-month depression history was more varied. The overall trend is such that higher conformity tended to be associated with a lower likelihood of reporting a 12-month depression history. However, differences were present across groups and this trend was not present for those aged 51–55 years.

**Table 3:** Influence of age and conformity to masculine norms on different indices of depression

	Currently depressed^a^			12-month depression^b^		
	OR^c^	*p* value	95% CI	OR	*p* value	95% CI
(Intercept)	0.17	**0.00**	[0.12, 0.23]	0.30	**0.00**	[0.23, 0.40]
Age						
15–17	1.61	0.15	[0.84, 3.10]	0.65	0.21	[0.33, 1.28]
18–25	2.03	**0.00**	[1.29, 3.18]	0.84	0.43	[0.55, 1.29]
26–35	1.39	0.13	[0.91, 2.12]	0.86	0.40	[0.60, 1.23]
36–50	1.05	0.80	[0.72, 1.53]	0.93	0.66	[0.69, 1.26]
15–17						
Moderate non-conformity^d^	0.88	0.70	[0.45, 1.72]	0.71	0.39	[0.33, 1.54]
Moderate conformity	0.62	0.61	[0.32, 1.20]	0.42	**0.03**	[0.19, 0.90]
Extreme conformity	1.22	0.60	[0.62, 2.37]	0.45	0.07	[0.19, 1.05]
18–25						
Moderate non-conformity^d^	0.70	0.08	[0.48, 1.04]	0.69	0.08	[0.45, 1.05]
Moderate conformity	0.98	0.89	[0.68, 1.40]	0.59	**0.01**	[0.39, 0.88]
Extreme conformity	1.03	0.88	[0.70, 1.53]	0.59	**0.03**	[0.37, 0.94]
26–35						
Moderate non-conformity^d^	0.77	0.12	[0.55, 1.07]	0.73	**0.05**	[0.53, 1.00]
Moderate conformity	0.95	0.77	[0.70, 1.31]	0.64	**0.01**	[0.47, 0.88]
Extreme conformity	1.37	0.09	[0.96, 1.97]	0.64	**0.03**	[0.43, 0.95]
36–50						
Moderate non-conformity^d^	1.18	0.17	[0.93, 1.49]	0.84	0.09	[0.69, 1.03]
Moderate conformity	1.31	**0.02**	[1.04, 1.64]	0.80	**0.03**	[0.65, 0.98]
Extreme conformity	1.70	**0.00**	[0.28, 2.28]	0.77	0.08	[0.58, 1.03]
51–55						
Moderate non-conformity^d^	1.36	0.12	[0.92, 2.01]	0.84	0.31	[0.61, 1.17]
Moderate conformity	1.26	0.27	[0.84, 1.87]	0.84	0.31	[0.60, 1.18]
Extreme conformity	2.45	**0.00**	[1.48, 4.06]	1.41	0.15	[0.89, 2.24]

Bold values denote statistical significance at the* p* < 0.05 level

All models adjusted for Index of Relative Social Disadvantage (IRSD) and Remoteness Area

^a^Based on published cut-off scores indicating clinically significant symptoms (PHQ-9 scores ≥ 10)

^b^Based on self-reported 12-month history of depression

^c^OR relative to the 51–55 group within CMNI category

^d^Reference category is extreme non-conformity

## Discussion

Recent years have seen an increase in research exploring the relationship between masculine norms and mental health [5]. However, population-based research is lacking, and few studies have purposefully considered the impact of age on adherence to masculine norms, and the extent to which mental health problems like depression are implicated as a result of higher conformity. This is a significant oversight, particularly when considering the demographic ageing of the population both in Australia and internationally [40]. Accordingly, this study aimed to address this gap by exploring how age differences may impact on the relationship between conformity to masculine norms and current depressive symptoms, as well as self-reported 12-month depression history.

In the present study, overall degree of conformity to masculine norms decreased across the lifespan, with the likelihood of being in a higher conformity category decreasing with age. These findings support our hypothesis and are consistent with existing research [15, 22, 23] and theory [12, 14] demonstrating less rigid gender roles in older men. The results also revealed an overall effect of age on depressive symptoms with those aged 51–55 years reporting significantly lower depressive symptoms compared to all age groups except for those aged 36–50 years. These findings are consistent with population estimates among Australian males demonstrating a higher incidence of mental health problems in younger adults that attenuates with age [41].

Numerous studies have demonstrated that adherence to masculine norms can be both beneficial and detrimental to men’s health [8]. Thus, focusing solely on total CMNI scores can mask important insights into specific dimensions of masculinity that may be uniquely associated with depressive symptomology [42, 43]. Consistent with our hypothesis, adherence to masculine norms was shown to be differentially related to current depressive symptoms in a multivariable model that accounted for all CMNI factors. Our results show that in this broad population sample of Australian males, increased conformity to masculine norms emphasising the importance of work, dominance, risk-taking, heterosexual presentation, and status was associated with less depressive symptoms (although we acknowledge that some of these behaviours may negatively impact others and/or affect the quality of men’s relationships). Whilst the magnitude of these associations was negligible, these findings are somewhat inconsistent with those in a recent meta-analysis which reported that increased conformity to heterosexual presentation, dominance, risk-taking, and status was associated with poorer mental health outcomes, and that conformity to the masculine norm of primacy of work was unrelated to mental health outcomes [5]. This is likely due to methodological differences in the current study, including a large population sample as well as variation in relation to the mental health outcome measures. An additional difference of particular note is that the current findings were derived from a robust multivariable model that accounted for the *simultaneous* influence of all CMNI factors on depressive symptoms as opposed to considering them univariately. Therefore, these inverse effects are what emerge *after* accounting for the detrimental influence of factors such as self-reliance and winning and highlight that it is these particular domains of masculinity that are problematic. Failure to account for the multivariable influences of masculinity can lead to spurious conclusions regarding the effect of distinct aspects of this on mental health outcomes.

When considering interactions between specific masculine norms and age, some aspects of masculinity appear to be more problematic for men in terms of depressive symptoms at different stages of life. For example, the impact of high adherence to playboy on depressive symptoms was significantly stronger in younger males compared to older males. It is possible that younger men may feel increased pressure to demonstrate their manhood through their sexual presentation but may not be successful in practice. This could result in increased stress which in turn may confer risk for depressive symptoms. In contrast, adherence to norms such as winning, self-reliance, and power over women generally had a stronger association with depressive symptoms for older men, relative to younger age groups. The impact of these norms on depressive symptoms is consistent with previous findings demonstrating detrimental impacts on mental health and psychological help-seeking [5]. It is likely that strong adherence to power over women will negatively impact interpersonal relationships, while adherence to norms emphasising winning may impact on help-seeking which may be perceived as a sign of failure or loss of control [9, 44]. Thus, an inability to resolve internalised expectations around these norms as an individual ages may be particularly problematic in terms of depressive symptomology.

Out of all the CMNI factors, self-reliance had the strongest impact on depressive symptoms. This is particularly important to consider given the consistent association between self-reliance and poorer mental health outcomes, as well as research demonstrating increased adherence to this with older age [15, 22]. In addition, a recent study using Ten to Men data demonstrated that self-reliance is a significant risk factor for suicidal ideation [45]. The CMNI-22 assesses self-reliance in relation to help-seeking (e.g., “*I never ask for help*”*).* Thus, males who strongly endorse this norm may be less likely to reach out to family, friends, or professionals for help when experiencing mental health problems, increasing the risk of depression [8, 20]. It is plausible that ageing-related changes create greater gender role conflict with regards to self-reliance, presenting multiple challenges for older high conforming males.

Adherence to masculine norms also appears to influence how men acknowledge and respond to depression. In this study, higher conformity to masculine norms was associated with an increased likelihood of reporting clinically significant depressive symptoms in those aged 36–50 and 51–55 years. Thus, while younger males were more likely to be in the higher conforming categories, extreme conformity to masculine norms appears to be particularly problematic for older males. These findings support our hypothesis and are consistent with those by Rice and colleagues who demonstrated that the relationship between masculinity and depression increased with age [23]. In terms of self-reported 12-month depression history, increased conformity to masculine norms tended to be associated with a decreased likelihood of a study participant reporting that they had received treatment for, or experienced symptoms of, depression in the preceding 12 months. Interestingly, this association was not evident in those aged 51–55 years, and just exceeded (*p* = 0.08) the statistical significance threshold in those aged 36–50. Whilst one could speculate reasons for the discrepancy in results between current depression and 12-month depression as it relates to masculinity—such as a reluctance to access health services or poor insight regarding their symptomology—further research is required to adequately explain this difference.

### Clinical implications

The present findings have a number of important implications for men’s health. Notwithstanding some nuances between age groups, the overall trend of an inverse relationship between masculinity and self-reported 12-month depression history highlights the fact that higher conforming males are likely not receiving treatment for their depressive symptoms. While the present study did not examine attitudes toward help-seeking or mental health stigma, these findings suggest that masculine norms negatively impact help-seeking attitudes. This is consistent with previous studies demonstrating that increased adherence to masculine norms is associated with poorer attitudes toward help-seeking in general, as well as for mental health problems specifically [46]. It is also possible that when men do present in primary care their depressive symptoms may not be recognised because males may not describe their symptoms in a prototypical way, but rather as a pattern of externalised symptomology (e.g., [47, 48]). Furthermore, there may be reluctance to admit to experiencing depressive symptoms, such as sadness, that could be perceived as a sign of weakness [7]. In the oldest group aged 51–55 years, extreme conformity was not linked to a lower likelihood of reporting a 12-month history of depression. This might reflect older men engaging with the health-care system more frequently due to other concerns, possibly a result of chronological ageing challenges, thus the opportunity for the detection of depression may increase.

Improving our understanding of how adherence to masculine norms changes over the lifespan, as well as the impact this may have on mental health could help facilitate more effective psychological treatment and inform the development of mental health promotion programs and policies. Consideration of the ways in which mental health services could be targeted and delivered to high conforming males may help reduce men’s reluctance to engage with mental health treatment. In addition, clinicians working with high conforming males could help male clients develop more flexibility in relation to what it means to be a man [42], as well as how ageing may impact on perceptions of masculinity [18]. This may be particularly important for older males undergoing important life transitions [12].

### Limitations and suggestions for future research

We believe this study is the first to systematically examine the relationship between conformity to masculine norms and measures of depression across age groups using a population sample of males. Nevertheless, this study is not without its limitations. Notably, the use of cross-sectional data limits our ability to make any firm conclusions about how ageing impacts on conformity to masculine norms as group differences may reflect cohort effects rather than developmental effects. Future studies should explore longitudinal patterns in order to strengthen our understanding of how individuals change their gender role beliefs with increasing age. A further limitation relates to the age of participants. The oldest men in this study were aged 55 years and future research with chronologically older men is still needed to elucidate how ageing impacts on adherence to masculine norms and how this may relate to mental health difficulties. In addition, this study excluded males who were not sufficiently proficient in English, which may have implications for the representation of particular cultural groups.

Other limitations include the exclusive use of self-report measures, including the lack of screening and diagnosis of depression by a clinician. It is also important to acknowledge that the CMNI scale was developed in the United States and whilst frequently used with Australian samples, it may not have identified specific masculine norms relevant to the Australian context. In addition, the CMNI-22 total scale only has moderate internal consistency. However, the current study also examined the CMNI subscales which have been suggested to yield more meaningful results [43]. There are several versions of the CMNI, ranging from 11 to 94 items [33]. Most recently, a 30-item version was developed, with preliminary results demonstrating superior psychometric properties to previous versions [49]. Future research should consider the use of these longer scales to further validate these findings. Furthermore, it has been suggested that men who conform to masculine norms might exhibit depressive symptomology more readily through externalising symptoms that fall outside the current diagnostic criteria [47]. Thus, future studies should include multiple measures of depression, including measures assessing externalising symptoms to ensure a more inclusive assessment of depression amongst males [50].

## Conclusion

The findings from the present study provide important insights into the complex relationship between conformity to masculine norms and depression across the lifespan in the largest all-male, national cohort study to be conducted internationally to date. Specifically, this study demonstrated that while conformity to masculine norms declines with age, higher adherence to masculine norms appears to be significantly linked with depressive symptomology and mostly in older males aged 36-to-55 years. Furthermore, this study demonstrates that only some aspects of masculinity appear to be problematic in the context of men’s mental health. Furthering our understanding of the impact of ageing on conformity to masculine norms as well as the nuanced relationship between masculinity and mental health outcomes is an important step for improving the mental health of men across the lifespan.

## Supplementary Information

**Additional file 1.** APPENDIX A. **Supplementary Table S1**. Descriptive statistics for subscale scores on the conformity to masculine norms inventory (CMNI-22) and the PHQ-9 by age group.

## Data Availability

Information on accessing Ten to Men data is available at https://tentomen.org.au/data-access-and-usage. Copies of Wave 1 questionnaires, Wave 1 data books, and the Ten to Men Data User’s Manual are also available at that site.

## Abbreviations

ASGS: Australian Statistical Geography Standard
CMNI: Conformity to Masculine Norms Inventory
GLM: Generalised linear models
IRSD: Index of Relative Socioeconomic Disadvantage
PHQ: Patient Health Questionnaire
