# Supplementary material for: Associations between conformity to masculine norms and depression: age effects from a population study of Australian men
Source: BMC Psychol. 2021 Feb 19;9:32. doi: 10.1186/s40359-021-00533-6 (PMC7893732; doi:10.1186/s40359-021-00533-6)
Supplement: Supplementary file 1 — Additional file 1. APPENDIX A. Supplementary Table S1. Descriptive statistics for subscale scores on the conformity to masculine norms inventory (CMNI-22) and the PHQ-9 by age group. [file 40359_2021_533_MOESM1_ESM.docx]

|  | | | | | | | | Age group | | | | | | | |
| --- | --- | --- | --- | --- | --- | --- | --- | --- | --- | --- | --- | --- | --- | --- | --- |
|  | | 15-17 years | | | 18-25 years | | | 26-35 years | | | 36-50 years | | | 51-55 years | |
| Factor ^a^ | Mean | | *SD* | Mean | | *SD* | Mean | | *SD* | Mean | | *SD* | Mean | | *SD* |
| Work | 3.03 | | 1.30 | 2.87 | | 1.28 | 2.63 | | 1.24 | 2.52 | | 1.16 | 2.53 | | 1.13 |
| Playboy | 1.68 | | 1.45 | 1.83 | | 1.49 | 1.62 | | 1.37 | 1.53 | | 1.30 | 1.46 | | 1.28 |
| Self-reliance | 2.43 | | 1.22 | 2.68 | | 1.25 | 2.58 | | 1.15 | 2.58 | | 1.10 | 2.62 | | 1.10 |
| Winning | 2.52 | | 1.27 | 2.57 | | 1.22 | 2.55 | | 1.11 | 2.42 | | 1.02 | 2.29 | | 0.99 |
| Dominance | 2.43 | | 1.18 | 2.51 | | 1.12 | 2.59 | | 1.10 | 2.46 | | 1.06 | 2.35 | | 1.10 |
| Risk-taking | 3.00 | | 1.32 | 3.06 | | 1.30 | 2.89 | | 1.19 | 2.65 | | 1.13 | 2.47 | | 1.10 |
| Emotional control | 3.30 | | 1.44 | 3.23 | | 1.49 | 3.11 | | 1.36 | 3.16 | | 1.32 | 3.20 | | 1.29 |
| Heterosexual presentation | 3.54 | | 1.71 | 2.84 | | 1.67 | 2.76 | | 1.55 | 2.95 | | 1.54 | 3.09 | | 1.57 |
| Power over women | 1.16 | | 1.10 | 1.23 | | 1.06 | 1.26 | | 1.02 | 1.29 | | 1.00 | 1.28 | | 1.02 |
| Violence | 2.84 | | 1.45 | 2.79 | | 1.45 | 2.55 | | 1.46 | 2.28 | | 1.43 | 2.09 | | 1.44 |
| Status | 3.54 | | 1.10 | 3.52 | | 1.07 | 3.49 | | 1.01 | 3.23 | | 0.98 | 2.99 | | 1.04 |
| PHQ-9 | 4.32 | | 4.67 | 4.97 | | 5.18 | 4.57 | | 4.65 | 4.30 | | 4.71 | 4.11 | | 4.95 |
| ^a^ Each factor is scored from 0 (lowest conformity) to 6 (highest conformity). | | | | | | | | | | | | | | | |

APPENDIX A. Supplementary Table

S1. Descriptive statistics for subscale scores on the conformity to masculine norms inventory (CMNI-22) and the PHQ-9 by age group
